# Prevalence of Systemic Hypertension and Control of Systolic Blood Pressure in a Cohort of 14 Dogs with Adrenal-Dependent Hypercortisolism during the First Year of Trilostane Treatment or after Adrenalectomy

**DOI:** 10.3390/ani14030511

**Published:** 2024-02-03

**Authors:** Paula García San José, María Dolores Pérez-Alenza, Daniel Alonso-Miguel, Sandra González Sanz, Carolina Arenas Bermejo

**Affiliations:** 1 Department of Animal Medicine and Surgery, Veterinary Faculty, Complutense University of Madrid, 28040 Madrid, Spain; mdpa@ucm.es (M.D.P.-A.); danialon@ucm.es (D.A.-M.); 2 Veterinary Teaching Hospital Complutense of Madrid, 28040 Madrid, Spain; sgsanz.vet@gmail.com; 3 AniCura Hospital Veterinario Valencia Sur, 46460 Valencia, Spain; caroarenas10@hotmail.com; 4 VetCT Teleconsulting, Cambridge CB3 0FA, UK

**Keywords:** hypercortisolism, Cushing’s syndrome, adrenal tumors, adrenalectomy, trilostane, hypertension, blood pressure, dog

## Abstract

**Simple Summary:**

Cushing’s syndrome is a common disease in middle-aged and old dogs, characterized by steroid overproduction by the adrenal glands. This steroid excess can be caused by an adrenal tumor (adrenal dependent hypercortisolism) and is associated with systemic hypertension. In people with this disease, it is known that hypertension can be difficult to manage and, sometimes, several drugs are necessary to treat it. However, there are no studies focused on the changes in the blood pressure during treatment in dogs with adrenal-dependent hypercortisolism. In this study, 14 dogs with this disease were followed during the first year of medical treatment or during 1 year after surgery to remove the adrenal tumor (adrenalectomy), and the changes in their blood pressure were evaluated. We observed that blood pressure in medically treated dogs decreased during the study period using, in most cases, a single antihypertensive drug. Also, in those dogs surgically treated, blood pressure normalized three months after surgery, and in one case, antihypertensive medications could be reduced. Thus, when adrenalectomy is performed, it is important to carefully address blood pressure after the procedure as these dogs might require reductions in their antihypertensive treatment.

**Abstract:**

Hypercortisolism in dogs is frequently associated with systemic hypertension (SH). However, there are no studies evaluating the changes in systolic blood pressure (SBP) in dogs with adrenal-dependent hypercortisolism (ADH) during trilostane treatment or after adrenalectomy and their response to antihypertensive treatments. For this reason, the objectives of this study were to evaluate the changes in SBP in dogs with ADH during the first year of trilostane treatment or after adrenalectomy, the relation with clinical control of hypercortisolism and certain laboratory parameters, and the response to antihypertensive drugs. Fourteen dogs newly diagnosed with ADH were prospectively included and evaluated at diagnosis (T0) and 1, 3, 6, and 12 months after (T1, T3, T6, and T12, respectively). Dogs were classified as hypertensive (HT; SBP ≥ 160 mmHg) and non-hypertensive. In HT dogs, benazepril was considered as the first-line drug, and, if necessary, amlodipine was prescribed. The prevalence of SH at T0 was 79%, and it was reduced to 25% at T12. Blood pressure (BP) was not associated with disease control or selected laboratory parameters at any endpoint. Only 22% of dogs with SH needed more than one drug to normalize their SBP. In all dogs surgically treated that were HT at T0, BP normalized at T3.

## 1. Introduction

Hypercortisolism or Cushing’s syndrome (CS) is a common endocrine disease in middle-aged and old dogs, which is characterized by a chronic glucocorticoid overproduction by the adrenal gland cortex. The most common causes are an adrenocorticotrophic hormone excess (ACTH) due to a pituitary tumor (pituitary-dependent hypercortisolism, PDH) or a functional adrenocortical tumor that autonomously produces cortisol (adrenal-dependent hypercortisolism, ADH) [1,2].

It is well documented that systemic hypertension (SH) is a common complication of CS, which may be present in approximately 70–85% of people [3,4,5,6] and 31–86% of dogs at the time of diagnosis [7,8,9,10,11,12,13,14,15]. There are also some factors that have been associated with SH in dogs with CS, such as lower potassium concentrations, thrombocytosis, higher cortisol concentrations, or higher urinary protein to creatinine ratio [7,10,11,12,13,15].

It is described that SH can persist even after appropriate control of the disease, both in people and dogs with PDH treated with trilostane or mitotane [3,4,5,6,7,8,13].

Even years after a successful surgical treatment, 20–40% of patients with CS (including children and adolescents with this syndrome) can be persistently hypertensive. These findings have also been observed in people with CS treated with different drugs (e.g., ketoconazole, metyrapone, pasireotide, cabergoline); despite most of them likely providing some degree of blood pressure improvement, persistent hypertension is also very common [3,4,5,6,16,17,18,19,20,21,22,23,24,25,26,27,28].

As increased arterial rigidity and increased cardiovascular risk are both described in children and adults with CS years after successful surgical treatment, it is widely accepted that normalization of cortisol concentrations does not always eliminate the metabolic and vascular disturbances related to CS [3,4,5,6,17,18,19,29,30,31,32].

Multidrug therapy is usually necessary to manage SH in people with CS [3,4,5,6]. Systemic hypertension is usually treated using angiotensin-converting enzyme inhibitors (ACEIs) and angiotensin II receptor blockers (ARBs) as first-line drugs, and calcium channel blockers as second-line drugs [3,4,5,6]. Also, as lower serum potassium concentrations have been associated with SH, which is indicative of an apparent mineralocorticoid excess, treatment with a mineralocorticoid receptor (MR) antagonist is recommended [3,4,5,6,33,34].

In dogs with CS, there are only four studies that have evaluated the changes in SBP before and after treatment. Two of them included dogs with PDH treated with mitotane and showed that SBP decreased only in dogs with good control of the disease [7,9]. Another study evaluated the changes in SBP in dogs with PDH after hypophysectomy or during trilostane treatment; in this study, no differences in SBP were observed during the study period [8]. A recent study evaluated the changes in SBP and response to antihypertensive treatment in dogs with PDH during the first year of trilostane treatment. In this study, a significant reduction in SBP was observed during the study period, and changes in SBP were not correlated with the clinical control of hypercortisolism. Multidrug therapy is commonly needed to manage SH. Importantly, in this study, they found that some normotensive dogs at diagnosis developed SH during trilostane treatment.

The literature on SH in dogs with ADH is sparce. One study showed that of four dogs successfully treated with adrenalectomy, none were hypertensive at the time of post-surgical re-evaluation [7]. To our knowledge, there are no additional studies that have evaluated the changes in SBP in dogs with ADH after surgery, and there are no studies that have evaluated these changes during trilostane treatment nor the response of these dogs to antihypertensive drugs.

Thus, the aims of this study were to evaluate the prevalence of SH in dogs with ADH at diagnosis and the changes in SBP after adrenalectomy or during the first year of twice-daily trilostane treatment. Also, to evaluate the relationship between SH and clinical control of the disease, the CBC results (as thrombocytosis has been previously associated with SH in dogs with CS), potassium and cortisol concentrations at every time point, and the response of these dogs to antihypertensive treatments were considered.

## 2. Materials and Methods

Dogs newly diagnosed with ADH at the Veterinary Teaching Hospital Complutense between January 2015 and December 2022 were prospectively included in this study. In order to increase the sample size, cases from AniCura Hospital Veterinario Valencia Sur were also retrospectively included. In all cases, owners consented to the use of the data from their pets for research purposes.

Diagnosis of spontaneous hypercortisolism was performed following the 2012 ACVIM consensus for the diagnosis of hyperadrenocorticism [1]. Adrenal function tests were performed in dogs with compatible clinical signs and physical examination findings and in those dogs with an adrenal mass. Diagnosis was confirmed when at least two of the following tests were consistent with hypercortisolism: urinary cortisol-to-creatinine ratio (UCCR), ACTH stimulation test (ACTH-st), or low-dose dexamethasone suppression test (LDDST). Discrimination between adrenal-dependent hypercortisolism and ACTH-dependent hypercortisolism was based on ultrasonography findings, plasma endogenous ACTH concentrations, and/or LDDST results [35].

Owners were informed about treatment options (surgery vs. medical treatment) but, ultimately, the final decision was theirs. Thus, classification of the dogs as medically treated with trilostane (trilostane group, TG) and surgically treated (adrenalectomy group, AG) was not randomized. In all cases, after definitive diagnosis of ADH, trilostane treatment was prescribed every 12 h at a starting dose of 0.2–1.0 mg/kg/12 h (either as a short-term treatment before surgery in order to minimize potential complications associated with hypercortisolism, or as a life-long treatment). In dogs in the AG, glucocorticoid supplementation was provided after adrenalectomy and tapered down according to clinical signs and electrolyte concentrations during follow-up; in dogs unable to maintain electrolyte concentrations within the target range during the hospitalization period, fludrocortisone was additionally prescribed.

Dogs were evaluated at diagnosis (T0) and at 1, 3, 6, and 12 months after the beginning of trilostane treatment or after adrenalectomy (T1, T3, T6, and T12, respectively). In those dogs in the AG, an additional endpoint was considered one week before surgery (T0′).

Dogs with chronic kidney disease of International Renal Interest Society (IRIS) stage 3 or 4, previously treated with antihypertensive drugs or trilostane, and patients lost to follow-up on any of the visits were excluded. Death during the study period was not an exclusion criterion as long as it occurred after T1.

At T0, complete data from signalment, clinical signs, physical examination findings, and concurrent diseases were recorded. Body condition score (BCS) was also recorded, and dogs were classified as underweight (BCS ≤ 3/9), ideal body weight (BCS 4–6/9), or overweight (BCS ≥ 7/9).

At every visit, a complete clinical history and physical exam were performed and dogs were classified as well controlled (WC, if the owners believed that a complete resolution of clinical signs was achieved), moderately controlled (MC, if the dogs showed improvement but still subtle clinical signs were present), and poorly controlled (PC, if the owner believed that clinical signs of hypercortisolism were still present). In those dogs in the TG, an ACTH-st and serum electrolyte concentrations were also performed at every visit. Trilostane dose was modified based on the results of the ACTH-st in combination with clinical control of the disease, as perceived by the owner. Cortisol concentrations were measured by chemiluminescence immunoassay (Immulite^®^ 2000, Siemens Healthcare S.L.U, Madrid, Spain). In those dogs in the AG, only electrolyte concentrations were evaluated at all endpoints. Additionally, at T0 and at T12, a CBC was performed in all dogs.

Systolic blood pressure was measured at every endpoint following the 2018 ACVIM Guidelines recommendations for the measurement of SBP [36]. In this study, Doppler ultrasonography (Vettex^®^ Uni 900, 65 Huntleigh Diagnostics Ltd., Cardiff, UK) was the technique chosen. The dogs were positioned in sternal or lateral recumbency with minimum restraint and an 8 Mhz flat probe was placed between the carpal and metacarpal pad of the left forelimb. Cuff size was chosen to cover 30% to 40% of the limb circumference at the site of cuff placement [36]. At least 5 reliable measurements were obtained after an acclimation period and before performing any other procedure; SBP value used for this study was the mean of these 5 measurements. Systemic hypertension was considered as SBP ≥ 160 mmHg [36]. Dogs were classified as non-hypertensive (NHT, SBP < 160 mmHg) or hypertensive (HT, SBP ≥ 160 mmHg) and sub-classified according to the risk of target organ damage (TOD) as normotensive (SBP < 140 mmHg), pre-hypertensive (SBP 140–159 mmHg), moderately hypertensive (SBP 160–179 mmHg), and severely hypertensive (SBP ≥ 180 mmHg) [36]. Additional visits to re-evaluate SBP other than those previously mentioned were completed when necessary, following ACIM consensus recommendations to control SH [36,37]. Systolic blood pressure at T0 was recorded before any anti-hypertensive treatment was prescribed.

Antihypertensive treatment was prescribed based on ACVIM recommendations [37]. If signs of TOD were observed or SBP was equal or above 200 mmHg, antihypertensive treatment was started. If no signs of TOD were observed, dogs with SBP ≥ 160 mmHg were re-evaluated after one week. In these dogs, if SBP ≥ 180 mmHg, hypertension was treated. If SBP was between 160 and 179 mm Hg, these dogs were either treated with antihypertensive drugs or re-evaluated one month after, depending on the clinical judgment.

An angiotensin-converting enzyme inhibitor (ACEI) was chosen as first-line treatment based on a proposed treatment algorithm for people with CS [4] and also based on ACVIM guidelines [36,37]. Dogs with SH were initially treated with benazepril 0.25 mg/kg/12 h; this dose was increased as necessary up to 0.5 mg/kg/12 h. If SBP was still not normalized, amlodipine was added at a starting dose of 0.1–0.25 mg/kg/24 h and gradually increased when necessary up to a maximum of 0.5 mg/kg/24 h. If, at this point, SH was still present, hydralazine was added (0.2 mg/kg/12 h). Fifteen days after any modification of the antihypertensive treatment, SBP was re-evaluated. When clinical signs of hypotension (e.g., syncope, taquycardia, weakness) appeared concurrently with an SBP < 120 mmHg, antihypertensive treatment was decreased [36,37].

Statistical analyses were performed using computer software (IBM, SPSS statistics for Windows, v.25.0, IBM corp., Amornk, New York, NY, USA). Most of the variables were not normally distributed, and the sample size was relatively small; thus, non-parametric tests were preferred. To compare categorical non-repeated measures between groups, Fisher’s exact test and Chi-square tests were used (percentages were used to express the data). For comparisons of non-repeated measures between 2 different groups, Mann–Whitney U test was selected, and for those comparing more than 2 groups, Kruskal–Wallis test was used; results are presented as the median, range, and interquartile range (IQR expressed as quartile 1 (Q1) and quartile 3 (Q3)). Correlation between continuous variables was assessed with the Spearman’s rank correlation test. To compare categorical repeated measures between the different selected time points, McNemar’s test was chosen for variables with 2 different categories and, for those with more than 2 categories, the Friedman test (percentages were used to express the data). For comparisons of continuous repeated measured, the Wilcoxon signed-rank test was selected (median, range, IQR). When data from one individual were presented, each dog was identified with a D and a number from 1 to 14 following enrolment. Statistical significance was defined at a p<0.05 threshold.

## 3. Results

Thirty dogs were diagnosed with ADH during the study period, twenty at the Veterinary Teaching Hospital Complutense and ten at AniCura Hospital Veterinario Valencia Sur. Of these dogs, nine were lost for follow-up, as follow-up was conducted at their respective referring centers. One dog was excluded as it had chronic kidney disease IRIS stage III. Four dogs died during the perioperative period after adrenalectomy, and two died of unknown causes before the first follow-up visit. Thus, data from 14 dogs with ADH were finally included: 12 from the Veterinary Teaching Hospital Complutense and 2 from AniCura Hospital Veterinario Valencia Sur.

Of the dogs included, 9/14 (64%) were medically treated with trilostane (TG, trilostane treatment group), and 5/14 (36%) were surgically treated (AG, adrenalectomy group). In all cases, dogs were initially treated with trilostane at a median initial dose of 0.46 mg/kg/12 h (range = 0.27–1.0 mg/kg/12 h; IQR = 0.3–0.6 mg/kg/12 h).

Of the nine dogs included in the TG, five dogs died during the study. Two died between T1 and T3, one suddenly died at home, and one was euthanized because of dyspnea caused by pulmonary metastases of an adrenal carcinoma. One dog was euthanized between T3 and T6 due to a colonic carcinoma, and two dogs were euthanized between T6 and T12 because the owners perceived poor quality of life.

Of the five dogs included in the AG, one suddenly died at home between T6 and T12. The median time since diagnosis until adrenalectomy was 145 days (range= 15–300 days; IQR= 48–285 days). Four of the adrenal glands removed were histopathologically consistent with adenomas (80%) and one as a carcinoma (20%). After surgery, all dogs were treated with dexamethasone intravenously followed with prednisone orally after discharge. In all cases, glucocorticoid supplementation was discontinued before T3. Only one dog required mineralocorticoid supplementation after surgery, which was discontinued before T1.

### 3.1. Signalment, Clinical Signs, Physical Examination Findings, and Concurrent Diseases at T0

#### 3.1.1. Signalment

The median age of the 14 dogs included was 13 years (range = 8–17 years; IQR= 10–13.25 years), and the median weight was 10.7 Kg (range = 3.9–35.0 Kg; IQR= 6.7–16.8 Kg). Further, 4 dogs were neutered males (4/14, 29%), 8/14 (57%) were neutered females, and 2/14 (14%) were intact females. Purebred dogs represented 79% of the cases (11/14); breeds represented were Yorkshire Terrier (2/9), West Highland White Terrier (2/9), Cocker Spaniel (2/9), Shih Tzu (2/9), Boxer (1/9), Maltese (1/9), and Pomeranian (1/9).

There were no statistically significant differences in these variables between dogs in the TG and in the AG.

#### 3.1.2. Clinical Signs and Physical Examination Findings

The median duration of clinical signs before diagnosis was 5 months (range = 2–12 months; IQR= 2.7–8.2 months). Further, 11 of the 14 dogs showed polyuria/polydipsia (78%), 9/14 (64%) polyphagia, 8/14 (57%) a “potbellied” appearance, 7/14 (50%) panting at rest, 7/14 (50%) thin skin, and 7/14 (50%) had poor hair quality. None of the dogs had calcinosis cutis. No statistically significant differences were observed in these variables between dogs in the TG and dogs in the AG.

At T0, none of the dogs had a BCS ≤ 3/9, 8/14 (57%) had a BCS between 4/9 and 6/9, and 6/14 (43%) had a BCS of 7/9 or above. All dogs with a BCS ≥ 7/9 were in the TG; however, the difference was not statistically significant (*p* = 0.086).

#### 3.1.3. Concurrent Diseases

At T0, 1/14 dogs (7%) had chronic kidney disease IRIS stage II, 3/14 (21%) had mitral valve disease stage B1, 3/14 (21%) had a neoplastic disease (mammary adenocarcinoma, colonic adenocarcinoma and meibomian gland adenoma), 1/14 (7%) had leishmaniosis, and 1/14 (7%) and 1/14 (7%) had a chronic enteropathy. No difference was observed between the TG and the AG in the frequency of concurrent diseases in each group.

### 3.2. Prevalence of SH and Median SBP at the Different Endpoints

#### 3.2.1. All Dogs with ADH (TG and AG)

At T0, the median SBP was 179 mmHg (range = 140–255 mmHg; IQR = 159–211 mmHg), and the prevalence of SH was 79% (11/14). When classified according to the risk of TOD, 21% (3/14) of the dogs were prehypertensive, 29% (4/14) were moderately hypertensive, and 50% (7/14) were severely hypertensive. Complete data are available in Table 1.

No significant differences were observed in the prevalence of SH between the different endpoints. The prevalence of SH was higher at T0 (79%) than at T6 (27%), although this was not statistically significant (*p* = 0.059).

On the other hand, there was a significant difference between the median SBP at T0 compared to T1, T3, and T6.

#### 3.2.2. Dogs with ADH Medically Treated (TG)

Considering only those dogs in the TG, all of them were hypertensive at T0 (9/9), and the median SBP was 185 mmgHg (range = 160–225 mmHg; IQR = 170–222 mmHg). When classified according to the risk of TOD, 44% (4/9) were moderately hypertensive and 56% (5/9) severely hypertensive. Complete data are available in Table 2.

There was a significant difference between the prevalence of SH at T0 (100%) compared to T3 (43%; *p* = 0.046) and T6 (33%; *p* = 0.046). The median SBP was significantly higher at T0 compared to T1, T3, and T6 and was also significantly higher at T1 compared to T3.

#### 3.2.3. Dogs with ADH Surgically Treated (AG)

Considering only those dogs in the AG, 2/5 (40%) were hypertensive at T0, and the median SBP was 156 mmHg (range = 140–210 mmHg; IQR = 146–195 mmHg). When classified according to the risk of TOD, 3/5 (60%) were pre-hypertensive and 2/5 (40%) were severely hypertensive. No statistically significant differences were observed in the prevalence of SH or median SBP between any of the endpoints. Complete data are available in Table 3.

### 3.3. Differences between TG and AG at the Different Endpoints

The median SBP at T0 was not significantly different (*p* = 0.11) between dogs in the TG (185 mm Hg, range = 160–225 mmHg, IQR = 170–222 mmHg) and in the AG (156 mm Hg, range = 140–210 mmHg, 146–195 mmHg). However, the proportion of dogs with SH was significantly higher in those dogs in the TG (100%, 9/9) compared to those dogs in the AG (40%, 2/5) (*p* = 0.027).

No other differences were observed between TG and AG regarding the prevalence of SH or median SBP at the remaining endpoints.

### 3.4. Relationship between Blood Pressure at T0 and Data from Signalment, Clinical Signs, Physical Examination Findings, and Concurrent Diseases

#### 3.4.1. All Dogs with ADH (TG and AG)

When all dogs with ADH were considered (n = 14), there were no significant differences in the prevalence of SH at T0 and data from signalment, clinical signs, duration of these clinical signs prior to diagnosis, physical examination findings, or concurrent diseases at T0.

Median SBP was higher in dogs diagnosed with mitral valve disease stage B1 (215 mmHg; range = 210–255 mmHg; IQR = 212–235 mm Hg) than in those without this condition (170 mmHg; range = 140–230 mmHg; IQR= 158–182 mmHg). It was also significantly higher in dogs with poor hair quality (i.e., alopecia, hypotrichosis, and/or dull coat) (210 mmHg; range = 170–255; IQR= 19–222 mmHg) than in those with unremarkable hair (160 mmHg; range= 140–180 mmHg; IQR= 154–174 mmHg; *p* = 0.002). No other statistically significant differences were observed between the median SBP and the remaining variables.

#### 3.4.2. Dogs with ADH Medically Treated (TG)

As all dogs in the TG were hypertensive at T0, it was not possible to compare the prevalence of SH and data previously mentioned.

Regarding the median SBP, this was again higher in dogs with a poor hair quality (211 mmHg; range = 170–255; IQR= 185–230 mmHg) than in those with a normal hair coat (170 mmHg; range 160–178 mmHg; IQR= 165–174 mmHg) (*p* = 0.048). No other statistically significant differences were observed between the median SBP and the remaining variables.

#### 3.4.3. Dogs with ADH Surgically Treated (AG)

There were no statistically significant differences or correlations between median SBP or the prevalence of SH and variables related to signalment, clinical signs, physical examination findings, and concurrent diseases.

### 3.5. Relationship between Blood Pressure at the Different Endpoints and Control of the Disease Based on Clinical Signs

Considering all dogs with ADH (n = 14) at T1, 6/14 (42%) were WC, 4/14 (29%) were MC, and 4/14 (29%) were PC. At T3, 7/12 (58%) were WC, 4/12 (33%) MC, and only 1/12 (8%) was PC. At T6, 9/11 (82%) were WC and 2/11 (18%) were MC. Lastly, at T12, 6/8 (75%) were WC and 2/8 (25%) were PC.

There was no statistically significant correlation between clinical control of the disease at any time point and the prevalence of SH or the median SBP.

Of dogs in the TG, at T1, 1/9 (11%) was WC, 4/9 (44%) were MC, and 4/9 (44%) were PC. At T3, 2/7 were WC (29%), 4/7 (57%) were MC, and 1/7 (14%) PC. At T6, 4/6 (67%) were WC and 2/6 (33%) were MC, and, lastly, at T12, 2/4 (50%) were WC and 2/4 (50%) were PC.

Of dogs in the AG at T0, 2/5 (40%) were WC and 3/5 (60%) were MC. From T1 to T12, all dogs in the AG were WC according to clinical signs. Control of the disease was significantly better in the AG than in the TG at T1 (*p* = 0.006) and at T3 (*p* = 0.047).

Due to the small sample size, the relationship between blood pressure and control of the disease depending on the treatment group could not be evaluated. Complete data are available in Table 4.

### 3.6. Relationship between Blood Pressure at the Different Endpoints and Selected Laboratory Parameters

Platelet count was not correlated with SBP and was not significantly different between HT and NHT at T0 or at T12. This absence of relationship was observed in all groups.

Potassium concentrations were not correlated with SBP at any time point and were not significantly different between HT and NHT dogs.

ACTH-st and LDDST results were not correlated with SBP at T0 and were not significantly different between dogs HT or NHT. In dogs with ADH medically treated, in which ACTH-st was performed at every visit, no relationship was observed between cortisol concentrations and blood pressure at the remaining time points.

No significant differences were observed in any of the parameters studied between TG and AG at any time point.

### 3.7. Relationship between Blood Pressure and Death during the Study Period

Median SBP and prevalence of SH at T0, T1, T3, and T6 were not significantly different between survivors and non-survivors. Evaluation was not made at T12 as all the measurements correspond to dogs that completed the study period.

Time since diagnosis until death was not associated with SBP at T0.

### 3.8. Antihypertensive Treatment Prescription and Resolution of Systemic Hypertension

**Figure 1:**
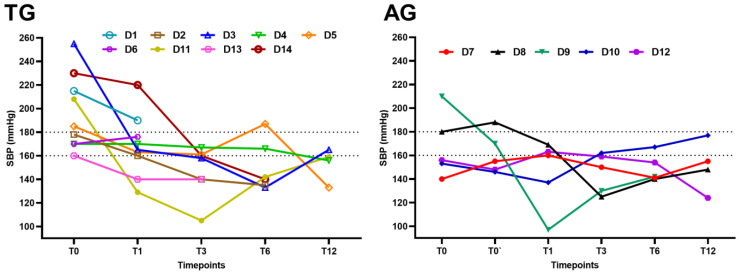
Systolic blood pressure of each dog (D) included in the study at the different endpoints. Trilostane group (TG) is represented on the left and adrenalectomy group (AG) on the right.

Measurements of SBP at each time point for each individual are represented in Figure 1. Antihypertensive treatment with benazepril was prescribed in 10/14 dogs (71%) during the study period. Further, 7 of these dogs (D) belonged to the TG (7/9; D1, D3, D4-D6, D11, D14) and 3 to the AG (3/5; D8-D10).

Two dogs needed additional treatment with amlodipine (2/9; 22%; D9, D14) and one of these two dogs needed a third drug to manage SH (i.e., hydralazine; D9). One belonged to the TG and one to the AG. Reductions in the antihypertensive medication were only possible in one dog from the AG, specifically D9. The dog was receiving combined treatment with hydralazine. Both amlodipine and hydralazine could be stopped after surgery, and the benazepril dose was also reduced, although it was not possible to interrupt it.

All dogs (9/9) in the TG were HT at T0. Resolution of SH was achieved in 7/9 cases: in two cases at T1 (one of these dogs had moderate hypertension at T0 and resolution was achieved without antihypertensive treatment; D11, D13), in two cases at T3 (D3, D5), in two cases at T6 (D2, D14), and in one case at T12 (D4). Two dogs died before resolution of SH was achieved despite antihypertensive treatment. The dog still HT at the end of the study period (D3) previously normalized his SBP; however, he was classified as being moderately hypertensive (165 mmHg).

Two dogs in the AG (2/5) were HT at T0. Resolution of SH in these dogs was achieved at T1 (D9) and at T3 (D8). In this group, there was still an HT dog at T12 (D10); this dog was NHT at T0.

## 4. Discussion

The prevalence of SH (SBP ≥ 160 mmHg) at diagnosis in dogs with ADH was 79%, which is slightly lower but similar to that previously reported in dogs with ADH [7,15] The prevalence of severe SH (SBP ≥ 180 mmHg) was 50%, similar to that observed in dogs with hypercortisolism (45%) [15] but more common than observed by others using ≥ 190 mmHg as the cut-off to define severe SH [7]. It is important to note that all of the dogs in the TG were HT but only 40% in the AG, which might have influenced the results of the study, especially when comparing the changes in the SBP between both groups.

Signalment, clinical signs at diagnosis, and physical examination findings were similar to those described in dogs with hypercortisolism [1,2,38,39]. Only poor hair quality (i.e., alopecia, hypotrichosis, and/or dull coat) was associated with an increased prevalence of SH and a higher median SBP when all dogs were considered and in dogs in the TG. In people, different types of alopecia (i.e., androgenic alopecia and frontal fibrosis alopecia) have been associated with SH, and early onset of this condition can even predict the development of SH. This also seems to be associated with higher aldosterone concentrations and with an increased arterial stiffness [40,41,42]. Our findings showed an unexpected association, as this has not been previously observed in dogs with CS [15]. The design of the study does not allow us to investigate this finding further; however, an increase in aldosterone secretion has not been demonstrated in dogs with ADH. Thus, the authors consider this potential physiopathological mechanism unlikely in our population [43]. It is also possible that this association simply indicates that dogs with an advanced stage of the disease involving hair abnormalities are more likely to be HT.

Regarding concurrent conditions, dogs with ADH and mitral valve disease were more likely to be HT than dogs without mitral valve disease. This has not been previously described in dogs with hypercortisolism [7,13,15]. It is important to note that an echocardiogram was not performed in all dogs as this was not part of the study design, so some of these dogs may have had stage A-B mitral valve disease. Previous studies have found that as many as 60% of dogs with hypercortisolism might have some degree of valvular disfunction [44]. To better evaluate the relevance of this association, further studies are needed.

To the authors’ knowledge, there are no previous studies evaluating the changes in SBP in dogs with ADH during treatment (either trilostane or adrenalectomy). Only one study has reported that all dogs (n = 4) with ADH were normotensive (SBP < 160 mmHg) after successful treatment with adrenalectomy [7]. In our study, the median SBP was significantly higher at T0 than at T1, T3, and T6, and the prevalence of SH decreased from 79% to 25% but this did not reach statistical significance. A decrease in the prevalence of SH was observed both for dogs medically treated, (from 100% to 25%) and for dogs surgically treated (from 40% to 25%). In dogs in the TG, this difference was statistically significant as there was a reduction in the median SBP during the study but not in the AG, probably due to the lower prevalence of SH at T0 in the second group. In dogs with ADH, the normalization of blood pressure seemed to be more common than in dogs with PDH, especially in those surgically treated, in which normotension was achieved at T3 in all dogs originally HT. Only one dog in the AG was HT after T3 (D10); this dog was originally NHT but, during surgery, complications associated with renal vein invasion of the adrenal mass occurred, and the vessel suffered some damage that was resolved. After surgery, this dog developed SH and remained HT until T12 despite normal renal function, as assessed by USG, SDMA, and creatinine. However, of note is that the prevalence of SH was still high at the end of the study in both groups (25%). This is similar to that observed in dogs with PDH 1 year after trilostane treatment, in which SH was 45% despite antihypertensive medication [13], and this is also seen in people with CS [3,4,5,6]. In people with CS, the metabolic and vascular changes persist even after successful medical treatment [3,4,5,6], which might be similar for dogs with ADH. However, BP normalized in all dogs in the AG that were HT at T0 at T3 until the end of the study period. Similar results were reported in a previous study in four dogs with ADH after surgical treatment, with all of them being normotensive at the time of evaluation [7]. In people with CS, blood pressure normalizes in 60–80% of cases after surgical treatment [5,20,21]. Similarly, in people with CS, the normalization of SBP seems to be more common after surgery in patients with ACTH-independent CS (specifically in cases of adrenal adenoma) than in patients with ACTH-dependent CS, likely because of incomplete pituitary tumor removal [20]. This might be similar to our observations, as in dogs in the TG, the cortisol source is not surgically removed. It is possible that in those dogs treated with trilostane, there is still some degree of chronic hypercortisolemia, which is unlikely in those dogs surgically treated as the cortisol-secreting tumor is completely removed. Interestingly, all dogs in the AG that were HT at T0 completely normalized their SBP at T3; this was the first endpoint in which there was no influence of glucocorticoids, as glucocorticoid supplementation could be discontinued in all cases before T3. However, the small sample size might have influenced the results, and this needs to be considered.

Clinical control of the disease was not associated with SBP at any time point. This differs to the results reported in dogs with PDH treated with a selective mitotane protocol, where an improvement in SBP was only observed in those dogs that achieved a good control of the disease based on ACTH-st results [7]. In the present study, control of the disease was based on the owners’ opinion, as previous studies have reported that the ACTH-st results in dogs under trilostane treatment do not seem to completely correlate with the clinical control of hypercortisolism. Despite this difference in the criteria to define control of the disease in comparison with the previous study, it is important to highlight that our results do not show a correlation between the ACTH-st results and SBP. We consider it more likely that the difference between the results from both studies reflects the different effects of trilostane and mitotane on the adrenal cortex as mitotane is an adrenocorticolytic drug, whereas trilostane is a reversible enzyme inhibitor [1,2]. In dogs on mitotane treatment, the ACTH-st results reflect the adrenal cortex reserve, which is considered normal in dogs with good control of the disease, whereas in dogs under trilostane treatment, it reflects the capability of trilostane to inhibit cortisol secretion but not the adrenal cortex reserve [1,2]. It is, therefore, likely that the ACTH-st results in dogs treated with mitotane are a better indicator of the degree of hypercortisolemia than in dogs under trilostane treatment and, therefore, may have a better correlation of its effects on SBP. As previously mentioned, ACTH-st results in dogs under trilostane treatment poorly correlate with clinical signs, and, at the moment of writing, there is no objective method to precisely monitor trilostane treatment [45,46,47]. The absence of correlation between clinical control of the disease in this study is similar to that previously described in dogs with PDH under trilostane treatment [13]. It must be considered that most dogs were treated with antihypertensive drugs, and, also, good clinical control of the disease was achieved in most dogs. This, added to the small sample size, did not allow us to compare the results between the TG and the AG, and it might also have led to type II errors.

No correlation was observed between SBP and platelet count, potassium concentrations, or ACTH-st results, conversely to that previously described in dogs with PDH under twice-daily trilostane treatment. In dogs with PDH, lower potassium concentrations and higher platelet count were associated with SH at diagnosis. Also, basal cortisol concentrations were associated with SBP during the follow-up [13]. This might reflect differences between dogs with PDH and ADH. However, it is possible that due to the small sample size and the high prevalence of SH at T0 (leading to a very small group of NHT dogs), this might have led to type II errors.

Benazepril treatment could not be discontinued in any dog in which it was prescribed. Only two dogs (22%) needed more than one drug to manage SH, which is lower than that observed in a previous study in dogs with PDH under trilostane treatment (42%) [13], and also lower than that reported in people with CS under ACEI treatment [48]. These results suggest that ACEIs (specifically benazepril) seem to be more effective to treat SH in dogs with ADH than in dogs with PDH; however, this finding should be further investigated in a larger sample of dogs. Antihypertensive treatment could be reduced only in one dog after adrenalectomy. Prior to surgery, the dog was treated with benazepril, amlodipine, and hydralazine, and the latter two could be discontinued after surgery. At T12, this particular dog (D9) was treated only with benazepril at a lower dose (0.35 mg/kg/12 h) than the one received before the surgery (0.52 mg/kg/12 h). The remaining dog in the AG with HT at T0 received a benazepril dose of 0.4 mg/kg/12 h that could not be reduced after surgery; however, normotension was achieved at T3 without any further antihypertensive drug. Our findings suggest that in dogs, the removal of a cortisol-secreting tumor leads to a rapid decrease in SBP and an improvement in SH; thus, after adrenalectomy, careful monitoring of blood pressure is advised as dogs under antihypertensive treatment might need readjustments in their medication.

The study has some limitations that need to be addressed. Due to the study design, it is not possible to fully evaluate the effects of either trilostane or adrenalectomy on SBP because, when needed, due to ethical reasons, dogs were treated with antihypertensive medication. It is also important to address that, at the moment of writing, no device has been completely validated to measure SBP in conscious dogs [36]; it is not possible to ensure that oscillometry would provide the same results. Also, as there is no objective method nowadays to monitor trilostane treatment, only clinical response was evaluated. In the present study, only benazepril or a combination of benazepril and amlodipine (and hydralazine if necessary) was used to treat SH, so other drugs such as ARBs might have led to different results. Finally, type II errors cannot be ruled out due to the small sample size.

## 5. Conclusions

This is the first study that evaluates the SBP during the first year of follow-up in dogs with ADH surgically or medically treated with trilostane and their response to antihypertensive treatment. Systemic hypertension is common in dogs with ADH. In dogs with ADH treated with trilostane, antihypertensive drugs seem apparently more effective to treat SH than in dogs with PDH, as multidrug treatment is less commonly needed. In dogs undergoing adrenalectomy, antihypertensive medication allowed for the normalization of SBP in all dogs that were originally HT. Thus, special care must be taken after adrenalectomy to avoid hypotension, as these dogs might need readjustments in their antihypertensive medication. In dogs with ADH, clinical control of the disease, platelet count, potassium concentrations, and cortisol concentrations do not seem to be associated with SBP during the first year of follow-up.

## Figures and Tables

**Table 1:** Prevalence of systemic hypertension, classification according to the risk of target organ damage (TOD), and median systolic blood pressure (SBP) at the different endpoints of all dogs with ADH. Letters indicate statistically significant differences.

Dogs with ADH (n = 14)					
	T0	T1	T3	T6	T12
**Number of dogs**	14	14	12	11	8
**Systemic hypertension (SBP ≥ 160 mmHg)**	11	10	5	3	2
	(79%)	(71%)	(42%)	(27%)	(25%)
**Classification according to risk of TOD**					
Normotension (SBP < 140 mmHg)	0	3	3	4	2
	(0%)	(21%)	(25%)	(36%)	(25%)
Pre-hypertension (SBP 140–159 mmHg)	3	1	5	4	4
	(21%)	(7%)	(42%)	(36%)	(50%)
Moderate hypertension (SBP 160–179 mmHg)	4	8	4	2	2
	(29%)	(57%)	(33%)	(18%)	(25%)
Severe hypertension (SBP ≥ 180 mmHg)	7	2	0	1	0
	(50%)	(14%)	(0%)	(9%)	(0%)
**SBP (mmHg)**					
Median	179 ^a,b,c^	163 ^a^	154 ^b^	142 ^c^	155
Range (Minimum–Maximum)	140–255	97–220	105–167	133–187	124–177
IQR (Q1–Q3)	159–211	140–171	132–160	140–166	136–163

^a^ (*p* = 0.014), ^b^ (*p* = 0.017), ^c^ (*p* = 0.029).

**Table 2:** Number of dogs with ADH medically treated with systemic hypertension (SH), sub-classification depending on the risk of target organ damage (TOD), and median systolic blood pressure (SBP) at the different endpoints. Letters indicate statistically significant differences.

Dogs with ADH Medically Treated (n = 9)					
	T0	T1	T3	T6	T12
**Number of dogs**	9	9	7	6	4
**Systemic hypertension (SBP ≥ 160 mmHg)**	9 ^a,b^	7	3 ^a^	2 ^b^	1
	(100%)	(78%)	(43%)	(33%)	(25%)
**Classification according to risk of TOD**					
Normotension (SBP < 140 mmHg)	0	1	1	4	1
	(0%)	(11%)	(14%)	(67%)	(25%)
Pre-hypertension (SBP 140–159 mmHg)	0	1	3	0	2
	(0%)	(11%)	(43%)	(0%)	(50%)
Moderate hypertension (SBP 160–179 mmHg)	4	5	3	1	1
	(44%)	(56%)	(43%)	(17%)	(25%)
Severe hypertension (SBP ≥ 180 mmHg)	5 ^c^	2	0 ^c^	1	0
	(56%)	(22%)	(0%)	(17%)	(0%)
**SBP (mmHg)**					
Median	185 ^d,e,f^	165 ^d,g^	158 ^e,g^	141 ^f^	158
Range (Minimum–Maximum)	160–225	129–220	105–167	133–187	133–165
IQR (Q1–Q3)	170–222	150–183	140–161	135–166	144–162

^a^ (*p* = 0.046), ^b^ (*p* = 0.046), ^c^ (*p* = 0.024), ^d^ (*p* = 0.017), ^e^ (*p* = 0.018), ^f^ (*p* = 0.046), ^g^ (*p* = 0.028).

**Table 3:** Number of dogs with ADH surgically treated with systemic hypertension (SH), sub-classification depending on the risk of target organ damage (TOD), and median systolic blood pressure (SBP) at the different endpoints. Letters indicate statistically significant differences.

Dogs with ADH Surgically Treated (n = 5)						
	T0	T0′	T1	T3	T6	T12
**Number of animals**	5	5	5	5	5	4
**Systemic hypertension (SBP ≥160 mmHg)**	2	2	3	2	1	1
	(40%)	(40%)	(60%)	(40%)	(20%)	(25%)
**Classification according to risk of TOD**						
Normotension (SBP < 140 mmHg)	0	0	2	2	0	1
	(0%)	(0%)	(40%)	(40%)	(0%)	(25%)
Pre-hypertension (SBP 140–159 mmHg)	3	3	0	2	4	2
	(60%)	(60%)		(40%)	(80%)	(50%)
Moderate hypertension (SBP 160–179 mmHg)	0	1	3	1	1	1
		(20%)	(60%)	(20%)	(20%)	(25%)
Severe hypertension (SBP ≥ 180 mmHg)	2	1	0	0	0	0
	(40%)	(20%)	(0%)	(0%)	(0%)	(0%)
**SBP (mm Hg)**						
Median	156	155	160	150	142	151
Range (Minimum-Maximum)	140–210	146–188	97–169	125–162	140–167	124–177
IQR (Q1–Q3)	146–195	147–159	117–176	127–160	141–154	136–166

**Table 4:** Data of prevalence of systemic hypertension (SH) and median systolic blood pressure (SBP) depending on the clinical control of the disease at the different time points.

	**T1 (n = 14)**			**T3 (n = 12)**		
	**PC** **(n = 4)**	**MC** **(n = 4)**	**WC** **(n = 6)**	**PC** **(n = 1)**	**MC** **(n = 4)**	**WC** **(n = 7)**
**SH** **(SBP ≥ 160 mmHg)**	3	3	4	0	2	3
	(75%)	(75%)	(67%)	(0%)	(50%)	(43%)
**SBP (mmHg)**						
- Median	167	175	161	158	150	150
- Range (Min-Max)	140–176	129–220	97–169	-	105–167	125–162
- IQR (Q1–Q3)	152–173	144–205	137–163	-	122–163	135–160
	**T6 (n = 11)**			**T12 (n = 8)**		
	**PC**	**MC**	**WC**	**PC**	**MC**	**WC**
	**(n = 0)**	**(n = 2)**	**(n = 9)**	**(n = 2)**	**(n = 0)**	**(n = 6)**
**SH** **(SBP ≥160 mmHg)**	-	0	3	1	-	1
		(0%)	(33%)	(50%)		(17%)
**SBP (mmHg)**						
- Median	-	141	142	162	-	151
- Range (Min-Max)	-	140–142	133–187	159–165	-	124–177
- IQR (Q1–Q3)	-	-	140–166	-	-	133–156

n: number of animals, PC: poorly controlled, MC: moderately controlled, GC: good controlled, IQR: interquartile range (quartile 1 (Q1)- quartile 3 (Q3)). T1, T3, T6 and T12: 1, 3, 6, and 12 months after beginning trilostane treatment or after adrenalectomy.

## Data Availability

The data presented in this study are available on request from the corresponding author. The data are not publicly available due to the authors’ decision.
